# Predictors associated with ICU nursing workload in a sample of records collected before and during the first peak of the COVID-19 pandemic: An analytical study

**DOI:** 10.15649/cuidarte.4255

**Published:** 2025-12-18

**Authors:** Diana Isabel Cáceres Rivera, Luis Alberto López-Romero, Judy Paola Martínez Patiño, Claudia Consuelo Torres Contreras

**Affiliations:** 1 Facultad de Enfermería, Universidad Cooperativa de Colombia. Doctora en Biomedicina. Bucaramanga, Colombia. Email: dianai.caceres@ucc.edu.co Universidad Cooperativa de Colombia Bucaramanga Colombia dianai.caceres@ucc.edu.co; 2 Facultad de Ciencias de la Salud. Universidad Autónoma de Bucaramanga-UNAB. Magister en Epidemiología. Doctorado en Metodología de la Investigación Biomédica y Salud Pública. Bucaramanga, Colombia. E-mail: alberlop60@gmail.com Universidad Autónoma de Bucaramanga-UNAB Bucaramanga Colombia alberlop60@gmail.com; 3 Universidad de Santander UDES. Maestría en gestión de servicios de salud. Bucaramanga, Santander, Colombia. E-mail: judy-147@hotmail.com Universidad de Santander UDES Bucaramanga Colombia judy-147@hotmail.com; 4 Universidad de Santander. Facultad de Ciencias Médicas y de la Salud. Instituto de Investigación Masira, Bucaramanga, Santander, Colombia. E-mail: clau.torres@mail.udes.edu.co Universidad de Santander Bucaramanga Colombia clau.torres@mail.udes.edu.co

**Keywords:** Nursing, COVID-19, Workload, Critical Care, Enfermería, COVID-19, Carga de Trabajo, Cuidados Críticos, Enfermagem, COVID-19, Carga de Trabalho, Cuidados Críticos

## Abstract

**Introduction:**

In recent years, the workload of nursing professionals in intensive care units (ICUs) has been described. Identifying associated factors may contribute to improving nursing care planning.

**Objective:**

To determine predictors associated with nursing workload in ICU settings using a sample of records collected before and during the first peak of the COVID-19 pandemic.

**Materials and Methods:**

This was an analytical cross-sectional study. A total of 97 ICU patient records were included. Descriptive and multivariate analyses were performed using robust linear regression, with the primary outcome being workload measured with the Nursing Activities Score (NAS).

**Results:**

The mean age was 57.67 ± 17.78 years, and 68.04% (n=66) were men. Statistically significant differences were observed between the pre-pandemic period and the first peak of the pandemic for variables such as disease category, ICU type, Sequential Organ Failure Assessment (SOFA) score, and number of nurses (p<0.001). A difference in median NAS values was observed, with 60.85 (Q1–Q3: 51.8–68.25) during the pre-pandemic period, compared with 183.40 (Q1–Q3: 149.30–204.40) during the first peak of the pandemic (p=0.001).

**Discussion:**

The workload levels identified in this study are consistent with those reported in similar studies. However, the specific scenario examined has scarcely been described in the existing literature.

**Conclusion:**

The pandemic increased the nursing staff's workload threefold. A weak, direct linear correlation was identified between workload and SOFA score. The pandemic year and the presence of cardiopulmonary conditions were identified as workload predictors.

## Resumen

**Introducción:**

En los últimos años se han descrito la carga laboral de los profesionales de enfermería en unidades de cuidado intensivo. Identificar factores asociados puede contribuir a una mejor planificación del cuidado de Enfermería.

**Objetivo:**

Determinar los predictores asociados a la carga de trabajo en enfermería de UCI en una muestra de registros tomada antes y durante el primer pico de la pandemia de COVID-19.

**Materiales y métodos:**

Corte transversal analítico. Se incluyeron 97 registros de pacientes de Unidades de Cuidado Intensivo. Se realizó un análisis descriptivo y multivariado, empleando una regresión lineal robusta, cuyo desenlace principal fue carga de trabajo a través del Nursing Activities Score (NAS).

**Resultados:**

El promedio de edad fue 57,67± 17,78 y el 68,04% (n=66) eran mujeres. Se encontraron diferencias estadísticamente significativas prepandemia versus primer pico de la pandemia para variables como categoría de la enfermedad, tipo de UCI, puntaje SOFA y número de enfermeras, entre otros. (p<0,001). Se evidenció una diferencia en la mediana del puntaje del NAS 60,85(Q1:51,8- Q3:68,25) durante la pandemia versus 183,40(Q1:149,30-Q3:204,40) el primer pico, con una p=0,001.

**Discusión:**

El nivel de carga laboral identificado en el presente estudio es equivalente a lo descrito en otros similares. Sin embargo, el escenario evaluado ha sido poco descrito en la literatura actual.

**Conclusiones:**

La pandemia triplico la cargar laboral del personal de enfermería. Se identificó una correlación lineal, directa y débil entre las cargas de trabajo y el SOFA, el año de pandemia, el tener patologías cardio-respiratorio, como factores de predicción de la carga laboral.

## Resumo

**Introdução:**

Nos últimos anos, tem sido descrita a carga de trabalho que os profissionais de enfermagem em unidades de terapia intensiva podem vivenciar. A identificação dos fatores associados pode contribuir para um melhor planejamento da assistência de enfermagem.

**Objetivo:**

Determinar os preditores associados à carga de trabalho de enfermagem em UTI em uma amostra de registros obtidos antes e durante o primeiro pico da pandemia de COVID-19.

**Materiais e métodos:**

Estudo transversal analítico. Foram incluídos 97 registros (47 pré-pandêmicos e 50 durante o primeiro pico da pandemia) de pacientes internados em Unidades de Terapia Intensiva. Foi realizada análise descritiva e multivariada por meio de regressão linear robusta, tendo como desfecho primário a carga de trabalho mensurada pelo Nursing Activities Score (NAS).

**Resultados:**

A média de idade foi de 57,67 ± 17,78 e 68,04% (n = 66) eram mulheres. Diferenças estatisticamente significativas foram encontradas pré- pandemia versus o primeiro pico da pandemia para variáveis como categoria da doença, tipo de UTI, SOFA e número de enfermeiros, entre outras. (p < 0,001). Uma diferença foi evidente na mediana do escore NAS 60,85 (Q1: 51,8- Q3: 68,25) durante a pandemia versus 183,40 (Q1: 149,30-Q3: 204,40) no primeiro pico, com p = 0,00.

**Discussão:**

O nível de carga de trabalho identificado no presente estudo é equivalente ao descrito em estudos semelhantes. No entanto, o cenário avaliado tem sido pouco descrito na literatura atual.

**Conclusões:**

A pandemia triplicou a carga de trabalho da equipe de enfermagem. Uma correlação linear fraca, direta, foi identificada entre as cargas de trabalho e o SOFA, o ano da pandemia e ter patologias cardiorrespiratórias como preditores da carga de trabalho.

## Introduction

During the COVID-19 pandemic, nursing was one of the disciplines required to lead the care of patients with COVID-19, particularly in intensive care units (ICUs)^1^,^2^. This care encompasses management activities (related to material, human, and financial resources), clinical care (providing timely, comprehensive, and individualized patient care), and teaching and research (for personnel in training and for patient and family education)^3^,^4^. Collectively, these activities lead to a high workload, and in recent years, even before the pandemic, this workload has shown a consistent increasing trend^5^-^7^. In Colombia, it has already been demonstrated that more than half of a nursing shift may be dedicated to a single patient, with direct care activities occupying the largest portion of that time^8^.

Increased nursing workload has been described as being associated with several factors, including hospital stays exceeding 3 days and higher Acute Physiology and Chronic Health Evaluation II (APACHE II) scores, which classify disease severity in ICU settings. The increase has also been linked to admissions from surgical services and diagnoses such as trauma and emergency conditions^9^. Consequently, greater patient complexity and acuity result in a higher nursing workload, which in turn necessitates a larger number of nurses.

During the health crisis triggered by the pandemic, the number of ICU units increased while the number of professionals remained the same, resulting in a significant impact on performance, workload, work pressure, emotional burden, and deterioration of quality of life^10^,^11^. The increased nursing workload in caring for patients with COVID-19 is mainly attributable to the specific procedures required to manage severe hypoxemia, as well as continuous monitoring and oxygen titration for patients^12^.

This panorama shows the need to understand the actual workload of ICU nursing professionals to promote high-quality care processes under optimal working conditions for nursing staff. Accordingly, this study aimed to identify predictors associated with ICU nursing workload using a sample of records collected before and during the first peak of the COVID-19 pandemic.

## Materials and Methods

**Study Design**

This was an analytical cross-sectional study.

**Setting**

This study was conducted in an ICU in Bucaramanga, Colombia. Pre-pandemic data were collected between July and December 2018, and data from the first pandemic peak were collected between February and May 2021.

**Population and Sample**

Non-probability sampling was employed, yielding a total of 97 patients who were assessed using the Nursing Activities Score (NAS): 47 in the pre-pandemic period and 50 during the first peak of the pandemic. No formal sample size calculation was performed.

**Eligibility Criteria**

The study included patients aged ≥18 years, selected through nonprobability sampling, who had an ICU stay of ≥48 hours, any medical diagnosis, and a written medical order in the clinical record to remain or continue in one of the participating ICUs (mixed, medical, surgical, or cardiovascular units). Only patients awaiting transfer to a general ward or receiving palliative care were excluded. The number of patients evaluated corresponded to the total monthly discharges from each unit.

**Instrument and Measurements**

Workload was measured using the NAS as the dependent variable. The NAS is a widely used scale for estimating the average amount of time a professional nurse spends during a 24-hour morning shift. It comprises seven categories subdivided into 23 activities. Each category yields a score according to the activities included, which comprise basic activities (monitoring and control, laboratory tests, medication, hygiene procedures, care of drains, mobilization and positioning, support and care of relatives and patient, and administrative tasks), ventilatory support, cardiovascular support, renal support, neurologic support, metabolic support, and specific interventions^13^. According to the authors, the NAS should be interpreted as follows^14^:

• A score of 100% indicates the workload of one nurse for a 24-hour shift.

• Two patients scoring 50% each would require one full-time equivalent (FTE) nurse for the entire 24-hour shift.

• If an ICU totals 350 points in 24 hours, it requires the work of 3.5 nursing FTEs for that day.

The NAS was originally validated in 15 countries^14^ and is the most widely used instrument worldwide for measuring nursing workload. It has also been previously applied by the authors of this study in Colombia^8^,^9^.

For this study, the NAS was recorded by a trained nurse using a paper-based form. Measurements were performed at two time points: first in 2018 and then in 2021 during the first peak of the COVID-19 pandemic.

A specific questionnaire was used to measure the independent sociodemographic variables, including age, sex, marital status, educational level, socioeconomic status, occupation, and social security coverage. Additional variables of interest included the scores of the clinical predictors APACHE II and the Sequential Organ Failure Assessment (SOFA). Variables related to the patient’s health status were also considered, such as source of admission, diagnostic category, mortality, and length of hospital stay. Within this set, ICU-related variables were collected, including the total number of patients, the number of nurses per shift, the unit occupancy rate, and the ICU type.

**Data Collection**

Data were collected by three nurses who were duly trained by the principal investigator of the study. At the end of each shift, they interviewed the on-duty nurse and completed the paper-based data collection form. The authors had previously designed this form to collect sociodemographic, clinical, predictive, ICU-related, and NAS data. Clinical information concerning the patient’s condition was verified using the electronic medical record. The collected data were subsequently entered into a coded Excel file, where typographical errors were checked and corrected.

**Data Analysis**

After data cleaning, the database was imported into STATA version 14.0 for statistical analysis. A descriptive analysis was performed for sociodemographic, clinical, and health status variables, stratified by data collection period (pre-pandemic vs. first pandemic peak). Continuous variables were summarized as medians with interquartile ranges (Q1–Q3) or as means with standard deviations, depending on the distribution of the variables, as confirmed by the Shapiro-Wilk test and skewness-kurtosis tests (Sktest). In contrast, polytomous nominal variables were presented as absolute and relative frequencies.

Bivariate analyses were performed by data collection period (pre-pandemic versus the first peak of the COVID-19 pandemic). Nominal variables were compared using Pearson’s chi-square or Fisher’s exact test. Likewise, median values were compared using the Kruskal-Wallis test or Student’s t-test depending on the distribution of the variables.

Robust simple linear regression models were used to estimate the effect of each potential predictor on the NAS. Additionally, robust simple linear regression models were developed for each independent variable of interest identified from the literature and the investigators’ clinical expertise, including age, sex, patient’s source of admission, diagnostic category, APACHE II score, ICU length of stay, and data collection period. Spearman’s correlation coefficients were calculated for APACHE II versus SOFA and for NAS versus APACHE II.

Finally, a robust multiple linear regression model was developed using the NAS as the outcome. Sociodemographic variables such as gender and age, along with health status and ICU-related factors—including patients’ source of admission, ICU length of stay, diagnostic category, APACHE II score, data collection period, and SOFA score (both continuous and in quartiles)—were considered as potential primary predictors. A p-value <0.05 was considered statistically significant. All statistical tests were two-tailed. Data were analyzed using STATA 14.0 software^15^. The complete dataset is publicly available on Mendeley Data^16^.

**Ethical Considerations**

This study complied with Resolution No. 08430 of 1993 issued by the Colombian Ministry of Health and was classified as involving “less than minimal risk”^17^. All participants provided written informed consent. The study protocol was approved by the institutional ethics committee (Concept No. 022- 2018), Subcommittee on Bioethics, Minute No. 010, dated May 21, 2018, and adhered to national and international regulations governing research involving human subjects^18^.

## Results

The mean age of participants was 57.67 ± 17.78 years, and 68.04% (n=66) were male. Table 1 shows statistically significant differences in nursing workload between the pre-pandemic period and the first peak of the pandemic for the variables of education, occupation, patient’s source of admission, discharge destination, disease category, ICU type, SOFA score, and number of nurses.

**Table 1:** Comparison of sociodemographic and clinical characteristics of the population before and during the first peak of the pandemic

Characteristics	All n= 97 % (n)	Pre-pandemic (2018) n = 47 % (n)	During the first peak of the pandemic (2021) n = 50 % (n)	p-value
Age, years (mean ± SD)	57.67 ± 17.78	57.40 ± 17.84	57.92 ± 17.9	0.887¥
Sex				0.388Ɨ
Female	31.96 (31)	36.17 (17)	28.00 (14)	
Male	68.04 (66)	63.83 (30)	72.00 (36)	
Marital status				0.075Ɨ
Single	34.02 (33)	40.43 (19)	28.00 (14)	
Married	39.18 (38)	38.3 (18)	40.00 (20)	
Cohabiting	15.46 (15)	6.38 (3)	24.00 (12)	
Divorced	3.09 (3)	2.13 (1)	4.00 (2)	
Widow/widower	8.25 (8)	12.77 (6)	4.00 (2)	
Socioeconomic status				0.749Ɨ
Low	27.08 (27)	23.91 (11)	30.00 (15)	
Middle	63.54 (61)	67.39 (32)	60.00 (30)	
High	9.38 (9)	8.7 (4)	10.00 (5)	
Educational level				<0.001Ɨ
None	3.09 (3)	4.26 (2)	2.00 (1)	
Incomplete elementary school	12.37 (12)	21.28 (10)	4.00 (2)	
Completed elementary school	16.49 (16)	6.38 (3)	26.00 (13)	
Incomplete secondary school	11.34 (11)	17.02 (8)	6.00 (3)	
Completed secondary school	24.74 (24)	31.91 (15)	18.00 (9)	
Completed technical program	12.37 (12)	10.64 (5)	14.00 (7)	
Incomplete university education	2.06 (2)	4.26 (2)	0.00 (0)	
Completed university education	17.53 (17)	4.26 (2)	30.00 (15)	
Occupation				0.005Ɨ
None	32.99 (32)	48.94 (23)	18.00 (9)	
Student	2.06 (2)	0.00 (0)	4.00 (2)	
Employee	22.68 (22)	17.02 (8)	28.00 (14)	
Self-employed	27.84 (27)	21.28 (10)	34.00 (17)	
Unemployed	3.09 (3)	6.38 (3)	0.00 (0)	
Pensioner/Retired	11.34 (11)	6.38 (3)	16.00 (8)	
Source of admission				0.007Ɨ
Emergency room	38.14 (37)	21.28 (10)	54.00 (27)	
Surgery	3.09 (3)	4.26 (2)	2.00 (1)	
Hospitalization	12.37 (12)	21.28 (10)	4.00 (2)	
Referral	32.99 (32)	38.3 (18)	28.00 (14)	
Other	13.4 (13)	14.89 (7)	12.00 (6)	
Patient discharge destination				<0.001Ɨ
Morgue	30.21 (29)	34.78 (16)	26.00 (13)	
Home	5.21 (5)	2.17 (1)	8.00 (4)	
Hospitalization	36.46 (35)	56.52 (26)	18.00 (9)	
General care unit	20.83 (20)	6.52 (3)	34 (17)	
ECMO ICU	7.29 (7)	0.00 (0)	14 (7)	
Disease category				<0.001ƗƗ
Infectious	10.31 (10)	21.28 (10)	0.00 (0)	
Metabolic	3.09 (3)	6.38 (3)	0.00 (0)	
Central nervous system	7.22 (7)	14.89 (7)	0.00 (0)	
Circulatory	7.22 (7)	14.89 (7)	0.00 (0)	
Respiratory	61.86 (60)	21.28 (10)	100.00 (50)	
Gastrointestinal	3.09 (3)	6.38 (3)	0.00 (0)	
Connective tissue	1.03 (1)	2.13 (1)	0.00 (0)	
Trauma	5.15 (5)	10.64 (5)	0.00 (0)	
Intoxication	1.03 (1)	2.13 (1)	0.00 (0)	
Type of ICU				<0.001Ɨ
Intermediate	16.67 (16)	0 (0)	32.00 (16)	
Full-care ICU	83.33 (80)	100 (46)	68.00 (34)	
Days of ICU stay (median: Q1–Q3)	11 [7; 16.5]	11.5 [7; 17]	10 [7; 15]	0.432£
Mortality (yes)	29.90 (29)	34.04 (16)	26.00 (13)	0.387Ɨ
APACHE II (median: Q1–Q3)	10 [0; 18]	---	10 [0; 18]	---
SOFA (mean ± SD)	6.64 ± 4.17	8.13 ± 3.49	5.24 ± 4.29	0.005¥
Total of patients (median: Q1–Q3)	14 [12; 14]	14 [13; 14]	13 [10; 26]	0.971£
Nurses at time of assessment (median: Q1–Q3)	4.0 [4.0; 4.0]	4.0 [4.0; 4.0]	4.0 [4.0; 8.0]	<0.001£
Percentage of occupancy (median: Q1–Q3)	92.59 [76.92; 100]	100 [85; 100]	90.60 [74.07; 100]	0.079£
Patient/nurse ratio (median: Q1–Q3)	3.25 [3.0; 3.5]	3.5 [3.25; 3.5]	3.0 [2.5; 3.25]	<0.001£

SD: Standard deviation; Q: quartile; ICU: Intensive care unit; APACHE: Acute physiology and chronic health evaluation; ECMO: Extracorporeal membrane oxygenation; ƗChi-square test for categorical variables. ƗƗFisher’s exact test. ¥Student’s t-test. £Mann– Whitney U test.

The median NAS during the first peak of the pandemic was 183.40 (Q1: 149.30; Q3: 204.40), compared with 60.85 (Q1: 51.80; Q3: 68.25) in the pre-pandemic period (p=0.001; Table 2).

**Table 2:** Comparison of the overall and activity-based NAS before and after the COVID-19 pandemic

Characteristic	All Median (Q1–Q3)	Pre-pandemic (2018) Median (Q1–Q3)	First peak of the pandemic (2021) Median (Q1–Q3)	p-value
Overall NAS	92.75 [61.9; 184.5]	60.85 [51.8; 68.25]	183.40 [149.30; 204.40]	0.001
Monitoring and control	20.35 [16.60; 36.20]	16.60 [16.60; 20.15]	36.20 [36.20; 36.20]	0.001
Laboratory procedures	4.30 [4.30; 4.30]	2.15 [2.10; 4.30]	4.3 [4.3; 4.3]	0.001
Administrative and managerial tasks	5.60 [5.60; 5.60]	5.60 [5.60; 5.60]	5.60 [5.60; 5.60]	0.073
Hygiene procedures	12.30 [4.10; 20.60]	4.10 [2.05; 4.10]	20.60 [20.60; 40.60]	0.001
Care of drains	0.0 [0.0; 1.08]	0.0 [0.0; 0.90]	0.0 [0.0; 1.80]	0.021
Mobilization and positioning	17.90 [12.40; 34.90]	12.40 [5.50; 12.40]	34.90 [17.90; 34.90]	0.001
Support and care of relatives or patients	4.0 [0; 4]	0.0 [0.0; 2.0]	4.0 [4.0; 36]	0.001
Medication administration	4.20 [4.20; 57.40]	4.20 [4.2; 4.20]	57.40 [27.40; 57.40]	0.001
Ventilatory support	5.80 [2.20; 7.60]	2.30 [1.40; 5.40]	7.60 [5.80; 7.60]	0.001
Cardiovascular support	2.45 [1.20; 3.70]	1.20 [0.6; 2.45]	2.5 [1.2; 3.7]	0.001
Renal support	7.0 [7.0; 7.0]	7.0 [7.0; 7.30]	7.0 [7.0; 7.0]	0.017
Neurologic support	0.0 [0.0; 0.0]	0.0 [0.0; 0.0]	0.0 [0.0; 0.0]	0.143
Metabolic support	1.30 [1.30; 2.60]	1.30 [1.30; 2.80]	1.950 [1.30; 2.60]	0.578
Specific interventions	0.9 [0; 2.1]	0.0 [0; 0.9]	1.90 [0; 3.2]	0.001

NAS: Nursing Activities Score; Q: quartile; ⱡKruskal–Wallis test for comparison of medians.

**Figure 1:**
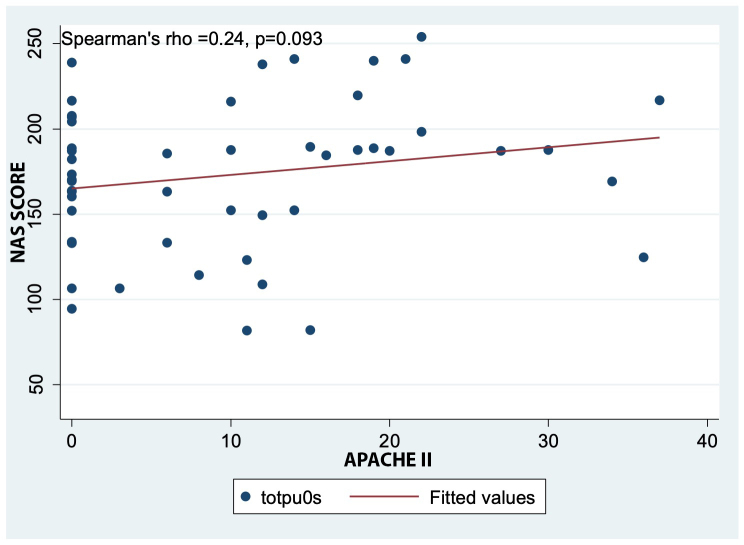
Spearman correlation between NAS and the APACHE II score

Figure 1 shows a direct but weak linear correlation between the NAS and APACHE II score. Although this association was not statistically significant (p=0.093), it is evident that as the probability of death increases, the nursing workload also increases.

**Figure 2:**
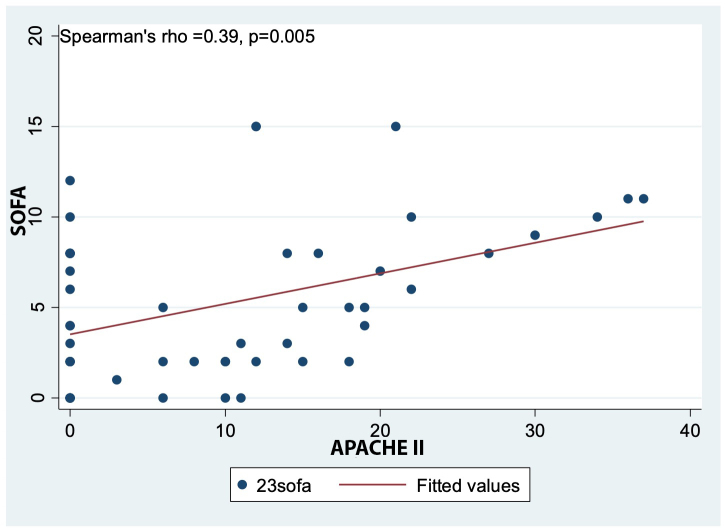
Spearman correlation between SOFA and APACHE II scores

Figure 2 shows a direct but weak linear correlation between the SOFA and APACHE II scores, with a statistically significant association (p=0.005). This indicates that as the SOFA score increases, the APACHE II score also tends to increase.

Regarding the robust linear regression model, the main findings indicated that the SOFA score, the pandemic year, the presence of cardiorespiratory conditions, and being a student or retired were associated with higher NAS. In other words, these variables are predictors of nursing workload (Table 3).

**Table 3:** Exploratory analysis of simple and fitted predictors of workload in a robust linear regression model

Nursing Activities Score (NAS)	Simple models: Raw effects			Final model (R2) (n=97)¥¥		
	β	(95% CI)	p value¥	β	(95% CI)	p value¥
Age (years)	0.30	(-0.42 to 1.03)	0.411	0.25	(-0.27 to 0.77)	0.339
β₀ = 101.56	R² = 65.06ⱡ	(60.21 to 142.90)	<0.001			
Sex		Reference: Women				
Sex (male)	0.56	(-28.52 to 29.63)	0.970	-9.77	(-23.24 to 3.69)	0.153
β₀ = 118.55	R² = 65.281	(93.96 to 143.14)	<0.001			
Occupation		References: None				
Student	81.06	(53.35 to 108.78)	<0.001	42.81	(11.68 to 73.95)	0.008
Employee	29.29	(-3.22 to 61.80)	0.077	2.62	(-16.34 to 21.58)	0.784
Self-employed	38.54	(6.24 to 70.84)	0.020	4.09	(-16.49 to 24.67)	0.693
Unemployed	-29.617	(-50.05 to -9.18)	0.005	3.14	(-14.88 to 21.14)	0.730
Pensioner/Retired	69.45	(21.10 to 117.81)	0.005	25.09	(3.79 to 46.38)	0.022
β₀ = 92.93	R² = 61.36	(73.61 to 112.26)	<0.001			
Source of admission		Reference: Emergency room				
Surgery	-35.06	(-124.81 to 54.69)	0.440	-10.07	(-28.89 to 8.76)	0.290
Hospitalization	-58.88	(-91.44 to -26.32)	0.001	5.60	(-11.32 to 22.52)	0.512
Referral	-22.37	(-53.06 to 8.32)	0.151	-0.36	(-15.05 to 14.32)	0.961
Other	-28.02	(-72.84 to 16.81)	0.218	-17.81	(-49.52 to 13.90)	0.267
β₀ = 138.44	R² = 63.43	(116.84 to 160.03)	<0.001			
Diagnostic category		Reference: Infectious-Metabolic				
Mental sphere	-3.14	(-15.65 to 9.36)	0.619	-5.08	(-25.90 to 15.74)	0.628
Cardiovascular	83.09	(66.64 to 99.54)	<0.001	-14.89	(-31.60 to 1.819)	0.080
Gastrointestinal	-1.92	(-16.67 to 12.84)	0.797	-2.91	(-33.13 to 27.29)	0.848
Trauma and emergencies	2.28	(-12.95 to 17.52)	0.767	5.61	(-14.25 to 25.47)	0.575
β₀ = 61.67	R² = 53.20	(55.77 to 67.55)	<0.001			
SOFA	-2.05	(-5.07 to 0.98)	0.183	2.48	(0.07 to 4.89)	0.043
β₀ = 132.52	R² = 64.72	(111.28 to 153.76)	<0.001			
Days of stay in ICU	-0.81	(-2.29 to 0.67)	0.281	0.14	(-0.83 to 1.11)	0.779
β₀ = 129.74	R² = 64.93	(106.62 to 152.86)	<0.001			
Type of ICU		Reference: Intermediate				
Full ICU	-35.71	(-60.64 to -10.78)	0.005	26.21	(-0.71 to 53.14)	0.056
β₀ = 149.35	R² = 63.89	(129.46 to 169.24)	<0.001			
Health care provider location		Reference: Pre-pandemic				
Post-pandemic	112.09	(99.32 to 124.86)	<0.001	133	(115.50 to 150.50)	<0.001
β₀ = 61.156	R² = 32.52	(57.75 to 64.56)	<0.001			

CI: Confidence interval ; ¥Robust simple linear regression model; ⱡRobust simple linear regression model;¥¥Linear regression model including age, sex, occupation, source of admission, diagnostic category, SOFA score, ICU length of stay, ICU type, and data collection period; *p-value of the adjusted robust multiple linear regression model.

Categorization of the SOFA scale showed that as the mean point of each SOFA quartile increased, workload measured by NAS remained approximately constant. Likewise, when comparing with the reference quartile (first quartile) in the robust multiple regression model adjusted for other factors, there was an average increase of 27 points in nursing workload across each subsequent SOFA quartile.

**Table 4:** Adjusted effect of categorized SOFA on workload as measured by NAS

SOFA quartile	Average SOFA quartile score X̄ ± SD	Minimum of SOFA quartile	Maximum of SOFA quartile	NAS mean score for SOFA quartile X̄ ± SD	Coefficient of linear regression† β, 95% CI
Quartile 1 (p25)	1.56 ± 1.19	0	3	140.04 ± 50.48	Reference category in regression analysis
Quartile 2 (p50)	5.34 ± 1.08	4	7	106.58 ± 65.00	27.06 (4.75–49.38)
Quartile 3 (p75)	8.81 ± 0.92	8	10	117.04 ± 70.95	27.00 (4.77–49.24)
Quartile (p100)	13.3 ± 1.95	11	17	111.56 ± 72.11	27.76 (-5.19–58.72)

SOFA: Sequential Organ Failure Assessment Score; NAS: Nursing Activities Score; SD: Standard deviation; CI: Confidence interval; ⱡRobust multiple linear regression adjusted for sex, age, occupation, source of admission, diagnostic category, ICU length of stay, ICU type, and data collection period.

## Discussion

This study aimed to identify predictors associated with ICU nursing workload using a sample of records collected before and during the first peak of the COVID-19 pandemic.

During the pandemic, a considerable increase in nursing workload was observed due to the intensive care required by patients diagnosed with COVID-19^19^. Our study analyzed clinical predictors associated with ICU nursing workload before and during the first peak of the COVID-19 pandemic; notably, NAS tripled during this period. Furthermore, although COVID-19 is a respiratory disease similar to many others, the nursing workload exceeded levels observed in patients with similar diagnoses, such as pneumonia of other etiologies^20^.

Among the main clinical predictors, higher APACHE II and SOFA scores were associated with higher NAS in ICU patients in this study. However, only the SOFA score demonstrated a statistically significant association. These findings have also been reported in ICUs from other countries. For example, the study by Bruynel et al. in Belgium reported a significant association between increased NAS among critically ill patients and elevated APACHE II scores (p=0.006). Other similar studies comparing NAS with APACHE II/IV scores have also shown statistically significant relationships^20^,^21^. This pattern could serve as a preliminary method to identify which patients will require longer periods of direct nursing care in units where no workload measurement tool has been established. Similarly, in older patients, higher age correlated with higher NAS, which may be related to their medical histories and clinical conditions, as advancing age increases the risk of complications in the ICU^8^,^22^.

Furthermore, we found that for every 25% increase in the SOFA score—which monitors a patient’s condition during their ICU stay—workload increased by an average of 27 points. This finding may guide the planning of nursing shifts and the implementation of contingency plans during periods of maximum patient flow or peak occupancy, such as pandemics or mass influxes of critically ill patients. Regarding disease category, few comparable studies were found. One of them, conducted in 2019, reported a significant difference between the type of admission and the nursing workload required by patients on the first day in the ICU (p=0.025)^23^. This finding was related to the patient acuity indicator used in that study, the Simplified Acute Physiologic Score (SAPS), which makes it non-comparable with our study. However, both studies indicate that there is no relationship between the patient’s source of admission and the NAS, although our study included a larger number of admission sources.

In the robust simple linear regression model, an association between the clinical characteristics of the patients included in the study and workload was found, particularly with cardiovascular patients and length of stay. These findings are consistent with a Brazilian study reporting moderate correlations between workload, length of stay, vasoactive drug use, and patient acuity^24^. Another Brazilian study involving 509 patients reported higher workloads for morning shifts, male patients, medical treatments, and patients admitted from emergency departments or other ICUs. Additionally, female nurses, a greater number of assigned patients, and longer ICU stays were directly associated with higher NAS. Work in surgical and burn ICUs has been inversely correlated with NAS^25^.

The number of nursing professionals was identified as a predictor associated with workload. This finding not only indicates the need for a greater number of professionals in critical care areas but also underscores the importance of ensuring that these professionals possess the necessary clinical competencies to manage the care of critically ill patients effectively. In such contexts, where the severity of the patient’s condition requires constant monitoring and rapid, accurate decision-making, nursing competence cannot be limited to technical skills alone; it must also encompass the ability to establish a strong and trusting therapeutic relationship with patients and their families. A relationship grounded in effective communication and emotional support is essential for reducing patient anxiety, enhancing care experience, and increasing adherence to treatment. Nurses who maintain consistent, empathetic interactions with their patients can detect earlier signs of complications and changes in their clinical condition, which is vital for timely intervention and improved clinical outcomes^26^.

Measuring workloads is therefore crucial to ensure that the number of available nurses is appropriate, enabling them to manage both the technical and emotional aspects of care properly. When nurses are overburdened, their ability to provide comprehensive, personalized care is compromised, directly affecting the quality of care provided. Consequently, the nurse-patient relationship becomes weakened, vigilance decreases, and the time available to address patients’ emotional needs is reduced. Thus, proper workload planning not only ensures that patients’ technical needs are met but also facilitates humanized, person-centered care, resulting in improved health outcomes and increased patient satisfaction. Ultimately, ensuring an adequate number of nurses, possessing the necessary clinical competencies, together with appropriate workload management, are key factors for delivering effective, safe, and empathetic critical care; these elements have a positive impact on patient outcomes and the overall quality of healthcare services^27^.

The period corresponding to the first peak of the pandemic had a clear impact on nursing workload, as confirmed by the final linear regression model. A similar finding was reported in a study from the Netherlands, which compared data from COVID-19 and non-COVID-19 periods and found significantly higher NAS among patients with COVID-19 than among those with pneumonia or without COVID-19^28^. The increased workload was likely due to more intensive hygiene procedures, increased need for mobilization and positioning, greater involvement in supporting families, and heightened respiratory care needs.

Although not originally defined as a study objective, our findings confirmed that using workload measurement tools, such as the NAS, helps identify needs related to ICU work dynamics and operational functioning. In particular, NAS remains one of the most widely applied instruments for managing nursing human resources in critical care^29^.

To our knowledge, this is one of the first studies to compare nursing workload before and during the first peak of the pandemic—a period that posed significant challenges and considerable pressure on healthcare systems and their personnel. However, the study has limitations. The healthcare system conditions during the two periods were markedly different, and the personnel working in the institution may also have changed over time, potentially limiting comparability. Likewise, the diagnostic profile differed completely between the two periods, with the pandemic period characterized by an emergent respiratory infectious condition caused by a novel pathogen. Moreover, the absence of a formal sample size calculation may have limited the statistical power needed to detect certain associations that were present; therefore, a type II error cannot be ruled out. Finally, no advanced statistical analysis, such as propensity score matching, was performed to attempt to adjust for differences in patients’ characteristics. Future studies with larger sample sizes and conducted across multiple centers are needed, using the types of advanced statistical techniques mentioned to compare these historical periods.

## Conclusions

The COVID-19 pandemic resulted in a threefold increase in nursing workload compared with the pre-pandemic period. This study identified a direct but weak linear correlation between nursing workload and the SOFA score. The pandemic year and having cardiorespiratory conditions emerged as predictors of workload. These findings can support care management by informing decisions, such as determining the number of nurses per patient and the level of technical support that may be required. They also serve as an indicator of how the quality of care can be improved through individualized approaches tailored to patients and unit characteristics.
